# Organ-Specific Regulation of Systemic Aging: Focus on the Brain, Skeletal Muscle, and Gut

**DOI:** 10.3390/cells15020153

**Published:** 2026-01-14

**Authors:** Jie Fu, Chengrui Liu, Yulin Shu, Yuxin Jiang, Ping Li, Kai Yao

**Affiliations:** 1 Institute of Visual Neuroscience and Stem Cell Engineering, Wuhan University of Science and Technology, Wuhan 430065, China; 2 College of Life Sciences and Health, Wuhan University of Science and Technology, Wuhan 430065, China

**Keywords:** brain, muscle, gut, systemic aging, exercise

## Abstract

**Highlights:**

**What are the main findings?**

- This review systematically elucidates the regulatory mechanisms of three critical target organs—the brain, muscle, and gut—in organismal aging and analyzes how exercise mitigates the aging process by modulating these tissues.
- We propose a novel “local-to-global” regulatory model, positing that preserving homeostasis within these specific tissues is sufficient to orchestrate systemic anti-aging effects via neural, metabolic, and immune pathways.

**What are the implication of the main findings?**

- The study offers a conceptual advance that supports a paradigm shift in medical research, moving away from non-specific systemic treatments toward precise, organ-targeted interventions.
- These insights provide a critical theoretical rationale for developing novel therapeutic strategies that target organ-specific homeostasis to combat age-related multimorbidity and extend healthy longevity.

**Abstract:**

As global population aging accelerates, the growing burden of age-related diseases is driving a shift in medical research from single-disease treatment to interventions targeting the aging process itself. Organ-specific interventions have emerged as a promising strategy to modulate systemic aging. Among organs, the brain, muscle, and gut have attracted particular attention due to their central roles in neural regulation, metabolic homeostasis, and immune balance. In this review, we focus on these three key organs, systematically summarizing their roles and regulatory mechanisms in organismal aging and discussing how exercise influences the aging process by affecting these organs. Crucially, we propose a novel “local-to-global” regulatory model, positing that preserving homeostasis in these specific tissues is sufficient to orchestrate systemic anti-aging effects. This work represents a conceptual advance by providing the theoretical rationale to move beyond non-specific systemic treatments toward precise, organ-targeted interventions.

## 1. Introduction

Aging is a complex biological process determined by multiple intrinsic and extrinsic factors. It is characterized by cellular homeostatic perturbation, tissue functional deterioration, and organismal physiological dysregulation [1]. Conventionally, aging has been attributed to the aggregate deterioration of multiple tissues. However, emerging evidence supports a “local-to-global” paradigm, where aging is not a uniform decay but a hierarchical process driven by specific “node” tissues [2,3]. Crucially, regulatory hubs are distinct from passive tissues because they actively drive systemic aging. A true hub is defined by its ability to alter organismal lifespan through local perturbations, regulate distal organs via circulating factors or neural networks, and integrate systemic feedback. Within this framework, the brain, muscle, and gut are considered key regulatory hubs. The brain, acting as the central regulator of organismal physiology, not only processes cognition and emotion but also orchestrates systemic homeostasis in response to environmental stress. Brain aging implies not merely neurodegeneration but a systemic compromise in the organism’s adaptive capacity [4]. Skeletal muscle, functioning as the largest metabolically active tissue and the effector of locomotion, dictates systemic functional reserve and metabolic competence [5,6,7]. Muscle aging is directly linked to frailty, loss of functional independence, and metabolic decline in the elderly [8]. The gut, serving as a vital organ for immune defense, nutrient absorption, becomes compromised with aging. Age-related loss of gut barrier integrity is often the starting point for increased systemic low-grade inflammation and metabolic disorders, which in turn accelerate functional decline in distal organs [9]. Physical exercise acts as a key anti-aging intervention targeting the brain, skeletal muscle, and gut. By reshaping local tissue niches and optimizing metabolic homeostasis, it effectively attenuates age-related functional deterioration [10,11]. Despite these advances, current literature often examines these organs in isolation. This fragmentation limits our understanding of how distinct organs can act as independent drivers to govern organismal lifespan and healthspan. To address this gap, this review synthesizes the evidence supporting this “local-to-global” model. We first delineate the distinct mechanisms by which specific brain regions regulate aging. Subsequently, we examine how skeletal muscle governs this process, followed by an analysis of gut-mediated modulation. Finally, we evaluate the specific impacts of physical exercise on the brain, muscle, and gut tissues, respectively. Unlike prior reviews that predominantly focus on isolated organ pathology, this review offers a comprehensive synthesis of how local molecular events dictate systemic lifespan, providing a conceptual foundation for tissue-specific anti-aging interventions.

## 2. The Brain as a Central Regulator of Aging: Hypothalamic Homeostasis and Hippocampal Plasticity

As the master regulator for systemic aging, the brain integrates internal and environmental signals through complex neuroendocrine and neural circuits to maintain organismal homeostasis. The efficacy of such regulation is fundamentally dependent on the brain’s structural and functional integrity, which progressively deteriorates with age [12]. Aging-associated brain changes involve atrophy of tissues, alteration in neurotransmitters, and damage accumulation in the cellular environment. Studies have shown that the total brain volume decreases by approximately 5% per decade, with shrinkage accelerating after the age of 70 [13,14]. This structural decline is typically accompanied by synapse loss. Such loss compromises neural information processing and integrative capacity. In *Macaca mulatta*, the density of thin spines in the prefrontal cortex decreases by about 33% from young (5–10 years) to old (20–30 years) age [15]. The reduction in synaptic number further triggers molecular dysfunction and impairs synaptic plasticity. Specifically, age-related changes in NMDAR subunits reduce the speed and concentration of Ca^2+^ influx, and ultimately impairing LTP (long-term potentiation) [16,17]. Changes in the brain not only affect brain function locally but also exert systemic consequences. In aging *Drosophila*, F-actin accumulates in brain tissue and impedes autophagosome–lysosome fusion. This failure prevents cells from clearing damaged mitochondria and protein aggregates, ultimately driving the functional decline of neurons. Pharmacological or genetic reduction in F-actin levels in aged neurons not only improves learning and memory in aged flies but also preserves gut integrity and muscle proteostasis, thereby markedly prolonging lifespan and extending organismal healthspan [18]. While such molecular deteriorations may manifest ubiquitously, their translation into systemic aging is primarily mediated by specific central hubs. Therefore, this review prioritizes the hypothalamus, hippocampus, prefrontal cortex, and brainstem, which serve as distinct but critical hubs regulating systemic homeostasis and organismal lifespan.

### 2.1. Hypothalamus

**Figure 1:**
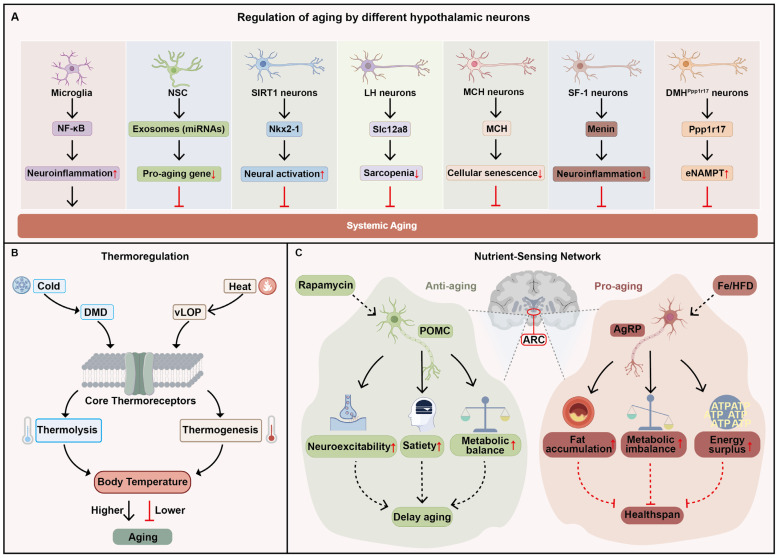
Mechanisms of hypothalamic regulation of systemic aging: (**A**) Distinct hypothalamic neuronal populations regulate systemic aging through multiple mechanisms; (**B**) The hypothalamus modulates systemic aging by regulating core body temperature; (**C**) In the arcuate nucleus (ARC), AgRP and POMC neurons sense nutrient status to govern the aging process. Black arrows indicate the direction of the process or activation, while red T-bars indicate inhibition. Red upward and downward arrows indicate increased and decreased levels, respectively.

As a higher-order regulator of aging, the hypothalamus counteracts age-associated pathophysiological changes and thereby promotes longevity [19]. Different hypothalamic cell populations regulate systemic aging via cell type-specific mechanisms in mice [20] (Figure 1A). Microglia, the brain’s resident immune cells, are critical regulators of neuroinflammation. Zhang et al. demonstrate that aging induces chronic activation of microglia in the mediobasal hypothalamus (MBH). This activation triggers persistent NF-κB signaling in adjacent neurons, which suppresses gonadotropin-releasing hormone (GnRH) expression. Inhibition of hypothalamic NF-κB signaling or exogenous GnRH supplementation markedly ameliorates aging-related phenotypes and extends lifespan [4,21]. Beyond inflammation-driven neuronal dysfunction, hypothalamic neural stem cells (htNSCs) also play crucial regulatory roles. In the mediobasal hypothalamus (MBH), htNSCs actively secrete exosome-packaged microRNAs (e.g., miR-17-5p and miR-146b-5p). With aging, levels of these htNSC-derived exosomal miRNAs are reduced in cerebrospinal fluid. This loss derepresses senescence-related genes, including *Cdkn2a* and *Txnip*, thereby accelerating cellular aging programs. Injection of exosomes derived from young htNSCs into aged mouse brains significantly alleviates cognitive decline and muscle atrophy [22,23]. Additionally, in the dorsomedial and lateral hypothalamic nuclei (DMH and LH), SIRT1-expressing neurons delay aging by deacetylating the transcription factor Nkx2-1 and thereby sustaining neural activation programs [24,25]. Moreover, a subset of LH neurons expressing the nicotinamide mononucleotide (NMN) transporter Slc12a8 regulate energy metabolism and skeletal muscle function through β2-adrenergic receptor-mediated sympathetic stimulation; restoring Slc12a8 expression in aged mice ameliorates sarcopenia and frailty-like phenotypes [26]. In parallel, in the LH, melanin-concentrating hormone (MCH) neurons protect against skeletal senescence through a specific neuroendocrine-PKA axis. Upon activation, these neurons secrete MCH into the circulation. The hormone then targets MCHR1 on bone marrow stromal cells to inhibit cellular senescence and drive osteogenesis, thereby countering age-related bone loss [27]. SF-1 neurons in the ventromedial hypothalamus (VMH) regulate aging through Menin. Its age-related decline triggers NF-κB mediated inflammation and suppresses D-serine production, thereby impairing cognition and shortening lifespan in mice. Notably, exogenous Menin supplementation in the aged hypothalamus effectively reverses these functional declines and systemic aging phenotypes [28]. However, in DMH, Ppp1r17 neurons regulate aging through hypothalamic-adipose communication. During aging, the PKG (protein kinase G)-mediated nuclear export of Ppp1r17 within DMH^Ppp1r17^ neurons increases, leading to reduced sympathetic drive to white adipose tissue, lowered lipolysis, and decreased EV (extracellular vesicle)-encapsulated eNAMPT (encapsulated extracellular nicotinamide phosphoribosyltransferase) secretion. Conversely, preventing the age-related nuclear export of Ppp1r17 or chemogenetically activating DMH^Ppp1r17^ neurons restores sympathetic output to WAT, enhances lipolysis and eNAMPT secretion, and extends lifespan [29].

Beyond neuron-specific pathways, hypothalamic thermoregulation constitutes an additional mechanism shaping organismal aging. Body temperature, a key determinant of physiological homeostasis, significantly influences the pace of aging. Chronically elevated core temperature markedly shortens mouse lifespan, whereas enhancing heat dissipation to reduce core temperature effectively rescues lifespan [30]. As the central hub for thermoregulation, the hypothalamus integrates thermal cues: heat activates neurons in the ventrolateral preoptic area (vLPO) to trigger cooling responses, while cold activates neurons in the dorsomedial dorsal subdivision (DMD) to elicit thermogenic warming [31] (Figure 1B). Accumulating evidence indicates that hypothalamic regulation of core body temperature can modulate aging processes and lifespan. Conti et al. report that hypothalamus-specific overexpression of the uncoupling protein UCP2 reduces core body temperature by ~0.3–0.5 °C and significantly extends lifespan in males (by ~12%) and females (by ~20%) [32]. The preoptic area (POA) of the hypothalamus is a torpor-regulating brain region. More recently, it has been found that activation of neurons in the POA induces a torpor-like state (TLS) characterized by hypothermia and hypometabolism without caloric restriction. Repeated induction of TLS robustly ameliorates aging-related phenotypes, as evidenced by lower frailty index scores, reduced tail rigidity, and improved gait. Furthermore, this intervention slows epigenetic aging and extends healthspan [33]. This discovery suggests a similar physiological regulatory mechanism may exist in hibernating animals. In fact, the convergent functional mutations observed in the hypothalamic cis-regulatory elements of various hibernating mammals indicate that these animals achieve a low metabolic, low inflammatory, and highly protective physiological state by modulating the set point for core body temperature [34].

Deregulated nutrient sensing is a hallmark of aging [35]. The nutrient-sensing neurons of the hypothalamus regulate aging by optimizing responses to nutritional availability in mammals [36,37,38]. The arcuate nucleus (ARC) is a pivotal nutrient-sensing center wherein orexigenic AgRP neurons and anorexigenic POMC neurons exert opposing control over appetite and energy homeostasis [39] (Figure 1C). POMC neurons release α-melanocyte-stimulating hormone (α-MSH) to suppress appetite and promote energy expenditure [40]. Compared to young mice, POMC neurons in aged mice exhibited a decrease in spontaneous firing and entered a state of functional silencing. This decline is linked to abnormal activation of the mTOR signaling pathway and diminished α-MSH secretion. Consequently, these changes lead to reduced satiety signaling and decreased energy expenditure in aged mice, resulting in obesity and metabolic dysregulation [41]. Intracerebral rapamycin, the mTOR inhibitor, infusion into old mice restores the excitability of POMC neurons, thereby reducing body weight and rescuing aging phenotypes [42]. As for AgRP neurons, these neurons promote appetite and suppress energy expenditure by releasing AgRP. AgRP antagonizes the α-MSH signals from POMC neurons [43]. AgRP innervation onto POMC neurons increases markedly with age, which correlates with a progressive augmentation of inhibitory postsynaptic currents and a concomitant reduction in POMC firing rate [44]. Consistently, in aged mice, the hypothalamus, particularly the ARC, exhibits a significant increase in iron content. Excessive iron further promotes the expression of the AgRP gene and leads to age-related obesity. Administration of the iron chelator deferiprone via the intranasal route reduces iron accumulation in the hypothalamus of aged mice, resulting in the inhibition of AgRP neuron activity and a significant decrease in AgRP mRNA expression. Consequently, the body weight of aged mice is markedly reduced, along with a decrease in fat mass [45]. Moreover, it is observed that AgRP-deficient mice exhibit enhanced metabolic capacity and attenuated age-related obesity [46]. Under high-fat diet conditions, the lifespan of AgRP-deficient mice is even longer than that of wild-type mice [47]. In addition to AgRP, AgRP neurons also influence aging by modulating nutrient-sensing pathways. Caloric restriction (CR) is a robust lifespan-extending intervention [48]. It is reported that CR activates AMPK and inhibits mTORC1 within AgRP neurons, thereby upregulating autophagy and initiating cellular stress-resistance and repair programs to enhance energy efficiency and metabolic homeostasis [49,50]. Additionally, aging AgRP neurons inhibit thermogenesis and reduce energy expenditure through OGT/O-GlcNAc and mTORC1 signaling, resulting in a sustained energy surplus. The consequent lipotoxicity induces hypothalamic ER stress, which aggravates central insulin/leptin resistance and compromises neuronal function. Collectively, these metabolic dysfunctions promote chronic inflammation and hasten the aging process [4,51,52].

### 2.2. Hippocampus

**Figure 2:**
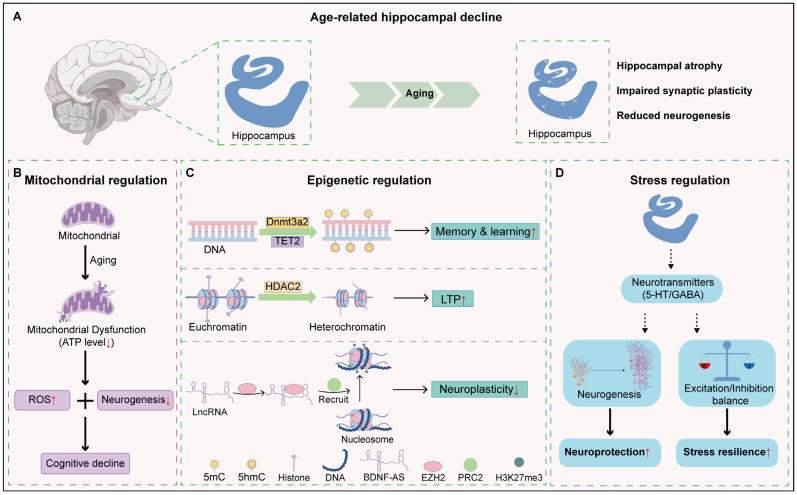
Key mechanisms by which the hippocampus mitigates systemic aging: (**A**) Changes in the hippocampus during aging; (**B**) Mitochondrial regulation of hippocampal aging; (**C**) The role of epigenetic regulatory factors in hippocampal aging; (**D**) Neurotransmitter dysregulation in hippocampal aging. Black arrows indicate the direction of the process or activation. Red upward and downward arrows indicate increased and decreased levels, respectively.

As a key hub integrating cognitive functions with the neuroendocrine system, the hippocampus is not merely a victim of aging, but an active participant in it. Single-nucleus transcriptome sequencing reveals that with aging, microglia and endothelial cells in the hippocampus display augmented inflammatory responses, which inhibit the neurogenic environment [53]. Through systematic analysis of more than 260,000 omics datasets, Hagihara and colleagues found hippocampal hyper-maturity in the mouse models of neuropsychiatric disorders. Further analyses revealed that hippocampal hyper-maturity shows accelerated aging-related transcriptional features with robust upregulation of aging hallmark pathways [54]. These molecular deficits manifest structurally as progressive volumetric atrophy, aberrant connectivity, and diminished neurogenesis (Figure 2A). Given the hippocampus relies heavily on mitochondrial ATP production for energy supply, mitochondrial dysfunction is an important driver of this decline [55,56]. Thus, preserving mitochondrial function is critical for delaying hippocampal aging and preventing related cognitive disorders (Figure 2B). Olesen et al. found that treatment of 18-month-old mice with the mitochondria-targeted antioxidant MitoQ or the natural compound curcumin markedly alleviates mitochondrial dysfunction in the hippocampus, restores energy metabolism, and improves learning and memory [57]. However, the mitochondrial mechanisms underpinning hippocampal dysfunction manifest significant heterogeneity across distinct pathological contexts. Ischemic stroke triggers acute mitochondrial collapse. Specifically, ischemia–reperfusion injury triggers a violent surge of oxidative stress, resulting in mitochondrial swelling, cristae fragmentation, and the lethal opening of the mitochondrial permeability transition pore (mPTP). The hippocampus initiates transient mitochondrial biogenesis as a compensatory measure during the acute phase. However, Bai et al. showed that this endogenous response is insufficient to bridge the massive metabolic gap required for neuroregeneration, leading to the developmental arrest of newborn neurons. Thus, repair requires the “forced reconstitution” of functional mitochondria. Notably, PGC-1α-mediated upregulation of the complex III subunit UQCRC1 drives mitochondrial biogenesis and respiratory recovery. This resolves metabolic bottlenecks, fuels dendritic remodeling, and reverses cognitive impairment [58]. Alzheimer’s disease (AD), conversely, involves a chronic imbalance in mitochondrial dynamics. As the disease advances, the pathological accumulation of Aβ and pTau, coupled with their deleterious interactions with mitochondrial regulators, disrupts the equilibrium of mitochondrial dynamics. Excessive fission impairs cristae integrity and energy production, while suppressed fusion leads to the accumulation of dysfunctional organelles. This disequilibrium ultimately culminates in widespread synaptic dysfunction [59]. Intriguingly, unlike its biogenic role in stroke, Wang et al. revealed that PGC-1α primarily regulates mitochondrial dynamics in AD. By promoting fusion and inhibiting fission, PGC-1α activates quiescent radial glia-like neural stem cells in the dentate gyrus. This optimization of mitochondrial dynamics promotes progenitor expansion and differentiation into mature neurons, ameliorating AD pathology and rescuing hippocampal-dependent cognition [60]. In Parkinson’s disease (PD), impaired mitophagy drives dopaminergic neuron death. Accumulated α-synuclein forms a “molecular barrier” blocking the clearance of damaged mitochondria. The resulting retention of defective mitochondria releases ROS and mtDNA, triggering energy crises and neuroinflammation that underlie progressive neuronal loss [61,62,63,64]. Crucially, this metabolic toxicity extends to the hippocampus. Since hippocampal mitophagy is fundamental to neurogenesis and memory, its failure in PD damages existing neurons and abolishes neurogenesis. This mechanism elucidates the clinical progression from motor dysfunction to cognitive decline [65,66]. Strikingly, mitochondrial transplantation offers a remedy. Zhang et al. showed that transplanting mitochondria isolated from young mice into the hippocampus of aged mice significantly boosts hippocampal neurogenesis and mitigates age-related cognitive decline [67]. Similarly, Zhao et al. demonstrated that intravenous delivery of young mitochondria to aged mice improves mitochondrial energy production, reduces oxidative stress, and markedly enhances cognitive performance [68].

During aging, epigenetic regulation within the hippocampus progressively deteriorates (Figure 2C). An increasing number of studies indicate that manipulating epigenetic factors may hold the key to delaying aging and enhancing the quality of life in older individuals [69]. DNA methyltransferase Dnmt3a2, which governs the expression of genes associated with synaptic plasticity, is essential for learning and memory consolidation [70]. Its expression declines markedly with age, leading to impaired epigenetic control in neurons. Restoration of Dnmt3a2 expression in the hippocampus significantly ameliorates age-related deficits in learning and memory, attenuates neuroinflammatory responses, and delays neural aging [71]. Similarly, the DNA demethylase TET2 plays a crucial role in sustaining neural stem cell function and adult neurogenesis. Age-related reductions in TET2 expression lead to a global decrease in 5-hydroxymethylcytosine (5hmC) levels and diminished neurogenic potential [72]. Restoration of hippocampal TET2 expression in aged mice elevates 5hmC at neurogenesis-associated loci. This epigenetic shift promotes neural stem cell proliferation and neuronal differentiation, and enhances cognitive performance, thereby reversing age-associated impairments in neuronal regeneration [73]. In addition, histone deacetylase HDAC2 accumulates within the aging hippocampus, repressing the transcription of synaptic plasticity-related genes and weakening long-term potentiation (LTP) and memory capacity. Deletion of HDAC2 effectively mitigates cognitive decline in aged mice [74]. Brain-derived neurotrophic factor (BDNF), a critical mediator of synaptic plasticity, supports neurogenesis, learning, and memory maintenance [75]. Its natural antisense transcript, BDNF-AS, is a long non-coding RNA transcribed from the antisense strand of the BDNF gene [76]. BDNF-AS interacts with the histone methyltransferase EZH2 to recruit the PRC2 complex to the BDNF promoter, facilitating repressive H3K27me3 modifications and suppressing BDNF transcription [77]. During aging, BDNF-AS-mediated repression of BDNF exacerbates the decline in hippocampal neurogenesis and accelerates cognitive deterioration. Genetic inhibition of BDNF-AS restores hippocampal BDNF expression, enhances transcription of neuroplasticity-related genes, and delays cognitive aging [77].

Another critical pathway through which the hippocampus modulates the aging process involves the regulation of neurotransmitter systems (Figure 2D). Significant alterations in GABAergic signaling constitute a primary driver of hippocampal aging [78]. Age-related declines in GABA synthase expression reduce neurotransmitter availability, triggering hippocampal hyperexcitability [79]. This imbalance is exacerbated by postsynaptic deficits, involving the downregulation of the GABA_A_ receptor α5 subunit and GABA_B_ receptors (notably in the CA3 region) [80]. Consistent with this receptor loss, electrophysiological recordings reveal a significant reduction in both the frequency and amplitude of GABA_A_ receptor-mediated inhibitory postsynaptic potentials (IPSPs) in the hippocampus of aged rats [81]. Collectively, these physiological and molecular impairments dismantle the hippocampal inhibitory network, thereby disrupting the encoding necessary for spatial memory formation and maintenance. Parallel to inhibitory deficits, the aging process critically impairs the synergy between the serotonin (5-HT) system and hippocampal neurogenesis. While neurogenesis—driven by neural stem cell proliferation—is fundamental for memory formation and adaptation [82,83], its capacity declines precipitously with age. This decline is mechanistically driven by a significant attenuation in serotonergic support. Specifically, aging compromises the system at both the source and target: a reduction in 5-HT neurons in the brainstem raphe nuclei leads to decreased projection density and 5-HT availability in the hippocampus [84]. This presynaptic deficit is compounded by postsynaptic alterations, where the expression and functional responsiveness of 5-HT1A receptors are diminished, rendering the hippocampus less sensitive to neurogenic signals. Consequently, this weakened serotonergic tone fails to sustain neural plasticity, directly contributing to cognitive dysfunction. In support of this perspective, findings from the Alzheimer’s Disease Neuroimaging Initiative (ADNI) have revealed a positive correlation between hippocampal serotonin (5-HT) levels and hippocampal volume. Elevated 5-HT signaling promotes the expression of BDNF, facilitates neurogenesis and synaptic plasticity, suggesting that targeting this pathway may help improve cognitive performance in individuals with Alzheimer’s disease [85,86].

### 2.3. Prefrontal Cortex and Brainstem

In addition to the hypothalamus and hippocampus, the prefrontal cortex (PFC)—the brain’s higher-order regulatory hub—plays multifaceted critical roles in modulating systemic aging. First, the PFC serves as a critical locus for the negative feedback regulation of the hypothalamic–pituitary–adrenal (HPA) axis, the central neuroendocrine system mediating stress responses and cortisol secretion. Research indicates that age-related atrophy of the PFC diminishes its inhibitory control over the HPA axis, resulting in chronically elevated cortisol levels. This prolonged elevation of cortisol, in turn, accelerates neurodegeneration in the PFC, establishing a deleterious positive feedback loop that drives systemic aging [87,88]. Beyond endocrine regulation, the PFC modulates systemic aging trajectories via the evolutionarily conserved regulation of neuronal excitability. Zullo et al. demonstrated that human longevity correlates closely with the downregulation of genes associated with cortical neuronal excitability and synaptic function. Specifically, compared to individuals aged 70–80, centenarians exhibit significantly higher levels of the nuclear transcription factor REST in PFC, exerting a more potent inhibitory effect on excitability-related genes. This mechanism has been validated in animal models. In aging mice, REST deficiency leads to aberrant cortical activity and neuronal hyperexcitability. Conversely, in *C. elegans*, inhibition of neuronal excitability via the REST homolog activates the conserved FOXO1/DAF-16 pathway, thereby extending lifespan [89].

Complementing the higher-order regulation by the PFC, the brainstem functions as a critical interface between the brain and the periphery, governing systemic aging through the modulation of the locus coeruleus-norepinephrine (LC-NE) system and the autonomic nervous system (ANS). The LC-NE system plays a central role in regulating cognition, neuroinflammation, and sleep homeostasis [90]. Specifically, norepinephrine (NE) signaling exerts neuroprotective effects by inhibiting NF-κB activation via the β_2_AR–cAMP–PKA pathway. However, age-related degeneration of LC neurons and the consequent reduction in NE release precipitate cognitive deficits. Pharmacological evidence supports this finding. Enhancing NE signaling suppresses glial-mediated inflammation and ameliorates cognitive decline in aging models [91,92]. Notably, structural atrophy of the LC disrupts sleep architecture. This brainstem-mediated fragmentation of sleep not only impedes the clearance of cerebral metabolic waste but also correlates with accelerated systemic aging and increased mortality risk [93,94,95,96,97]. Furthermore, as the central regulator of the ANS, the brainstem directly influences peripheral organ aging. In the rostral ventrolateral medulla (RVLM), glial senescence triggers local inflammation and sustained sympathetic overactivity, driving hypertension and cardiovascular aging [98,99,100]. Conversely, chronic stress activates the dorsal motor nucleus of the vagus (DMV). In this region cholinergic signaling induces cell cycle arrest in intestinal stem cells via the p38 MAPK pathway, thereby accelerating intestinal aging [101].

Collectively, the hypothalamus, hippocampus, PFC, and brainstem underscore the critical role of brain-derived regulation in maintaining organismal homeostasis. Nevertheless, systemic aging is a multi-tissue phenomenon that is also governed by distinct peripheral regulators. The following sections will examine the specific contributions of these peripheral organs, starting with skeletal muscle.

## 3. Regulatory Role of the Muscle in the Aging Process

**Figure 3:**
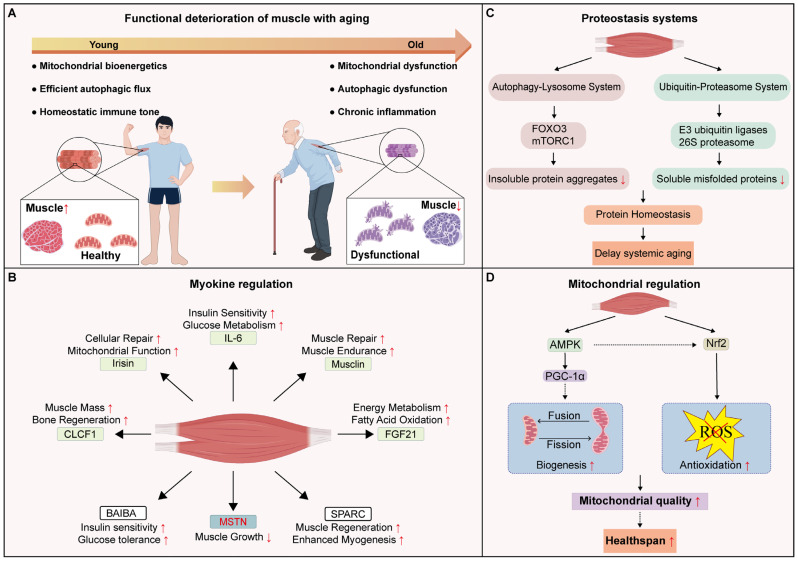
Skeletal muscle aging: Functional deterioration and systemic regulation mechanisms: (**A**) Aging induces muscle atrophy and functional decline; (**B**) Myokines improve systemic metabolic homeostasis and delay aging; (**C**) The autophagy–lysosome system (ALS) and the ubiquitin-proteasome system (UPS) work together to clear damaged proteins, preserving proteostasis and muscle homeostasis, thereby slowing systemic aging; (**D**) Skeletal muscle promotes mitochondrial biogenesis and antioxidant defenses via AMPK and Nrf2 signaling, improving mitochondrial quality and extending healthspan. Black arrows indicate the direction of the process or activation. Red upward and downward arrows indicate increased and decreased levels, respectively.

Skeletal muscle acts as a critical regulator of systemic aging, functioning not only as a locomotor organ but also as a vital metabolic and endocrine tissue. However, with advancing age, skeletal muscle progressively loses its structural integrity and functional capacity. This phenomenon is referred to as muscle aging (Figure 3A). Aging not only directly leads to reductions in muscle mass and strength but also imposes an increasing systemic burden on the organism. As muscle function deteriorates, motor performance declines markedly, resulting in a significantly elevated risk of falls and fractures [102]. Moreover, as a crucial tissue for maintaining energy metabolism, aged skeletal muscle exhibits impaired metabolic regulatory capacity, which contributes to the development of multiple metabolic disorders [103,104,105].

### 3.1. Endocrine Function and Metabolic Regulation

Skeletal muscle exerts its endocrine function primarily through the secretion of a variety of growth factors and cytokines. These molecules, collectively known as myokines, play critical roles in regulating energy metabolism and immune homeostasis [106] (Figure 3B).

As a prototypical myokine, Interleukin 6 (IL-6) acts as a context-dependent switch between metabolic regulation and inflammation. Physiologically, muscle-derived IL-6 orchestrates systemic metabolism to mitigate age-related metabolic dysregulation. It optimizes insulin sensitivity by upregulating glucose transporter 4 (GLUT4) and stimulating glucagon like peptide 1 (GLP-1) secretion from the gut. Additionally, IL-6 maintains lipid homeostasis by driving hepatic lipid clearance and sustaining adipos lipolysis [107,108,109,110,111,112,113,114]. However, aging triggers a maladaptive shift, turning IL-6 from a homeostatic metabolic regulator to a potent pro-inflammatory mediator. Mechanistically, this switch is driven by the age-associated downregulation of SIRT5, which induces the hypersuccinylation of TBK1 and activates the RelA signaling axis to exacerbate inflammaging [115].

Cardiotrophin-like cytokine factor 1 (CLCF1) belongs to the IL-6 family and is important for cardiac development and neuronal survival. Recent studies have shown that CLCF1 is also an exercise-induced myokine, the levels of which decline with age and can be restored through resistance exercise [116]. In skeletal muscle, CLCF1 improves muscle strength and endurance by promoting myogenesis and enhancing cellular metabolism. In bone, CLCF1 suppresses osteoclastogenesis through STAT1 and enhances osteoblast precursor differentiation via STAT3, leading to improved bone density and microstructural integrity. Administration of recombinant CLCF1 in aged mice reverses muscle atrophy, endurance loss, and bone deterioration, whereas CLCF1 knockout or blocking CLCF1 activity abolishes the anti-aging benefits of exercise [117].

Irisin, generated by proteolytic cleavage of its precursor FNDC5 under the control of PGC-1α, is another prominent exercise-induced myokine [118]. Muscle-derived irisin circulates systemically and exerts pleiotropic anti-aging effects largely mediated through AMPK activation [119]. In pancreatic β cells, this signaling inhibits NF-κB p65 phosphorylation to alleviate metabolic stress-induced glucolipotoxicity and senescence [120]. In vascular endothelial cells, it triggers the downstream Akt-eNOS cascade to mitigate age-related vascular dysfunction [121]. In cardiomyocytes, the pathway alleviates hypoxia-induced endoplasmic reticulum stress via PGC-1α to preserve mitochondrial function and attenuate senescence-associated damage [122]. Notably, in a mouse model of chronic cerebral hypoperfusion (CCH), exercise-induced irisin modulates the integrin αVβ5/AMPK/mTOR axis to attenuate CCH-related brain aging [123].

Fibroblast growth factor 21 (FGF21) is a stress-responsive myokine, induced by fasting, endoplasmic reticulum stress and mitochondrial dysfunction [124]. In mice, muscle-specific FGF21 overexpression enhances mitochondrial function and attenuates muscle atrophy [125]. Conversely, muscle-specific FGF21 deletion leads to spontaneous pulmonary inflammation and hepatic lipid accumulation [126]. Interestingly, clinical studies have revealed that in patients with amyotrophic lateral sclerosis (ALS), neurodegenerative changes and metabolic stress trigger a compensatory upregulation of FGF21 in atrophic muscle fibers. This upregulation protects both muscle cells and motor neurons by suppressing apoptosis and promoting myogenesis [127].

Musclin, also known as osteocrin (OSTN), is a muscle-derived metabolic regulator [128,129]. Kang et al. found that Musclin maintains muscle cell homeostasis by effectively inhibiting the proliferation of fibro-adipogenic progenitors (FAPs) and promoting their apoptosis. Furthermore, the supplementation of exogenous Musclin significantly promotes tissue regeneration, and ameliorates muscle damage [130]. In cancer cachexia, Musclin supplementation mitigates muscle atrophy and fibrosis, and enhances skeletal muscle resistance to injury [131]. In a mouse model of heart failure, muscle-specific overexpression of Musclin enhances cardiomyocyte contractility and attenuates myocardial fibrosis, thereby improving cardiac performance and delaying cardiac aging [132].

Myostatin (MSTN,/GDF-8), a TGF-β superfamily myokine, functions as a critical negative regulator of muscle mass [133]. Mechanistically, MSTN orchestrates a catabolic shift through the activin receptor type IIB (ActRIIB)-Smad2/3 signaling cascade, which suppresses protein synthesis and accelerates protein degradation [133]. Genetic models highlight the systemic benefits of MSTN inhibition. Aged MSTN heterozygous mice (*MSTN*^+/−^) exhibit preserved muscle function and extended lifespan, while knockout mice (*MSTN*^−/−^) are protected against diet-induced obesity and metabolic dysfunction [134,135]. Moreover, Deng et al. demonstrated that MSTN deficiency enhances thermogenesis, improves glucose metabolism and reduces chronic low-grade inflammation [136]. Mechanistically, this deficiency activates the AMPK-PGC-1α-FNDC5 signaling axis in skeletal muscle, which promotes browning of white adipose tissue and enhances mitochondrial function [137]. However, the role of MSTN exhibits evolutionary divergence. In *Drosophila*, muscle-specific knockdown of Myoglianin, a homolog of MSTN, shortens lifespan and exacerbates age-associated locomotor decline, whereas overexpression of Myoglianin exerts the opposite effect [138].

The aforementioned myokines represent several classes of myokines that have been relatively systematically and thoroughly researched to date. In addition to these classical molecules, several other myokines have also been implicated in the regulation of organismal aging (Table 1). In summary, skeletal muscle orchestrates systemic aging through a sophisticated myokine repertoire, where factors like Irisin, FGF21, and CLCF1 converge to sustain metabolic and regenerative homeostasis. However, this regulatory machinery is characterized by significant complexity, distinguishing the pleiotropic nature of mediators such as IL-6 from the evolutionary divergence observed in MSTN signaling. Consequently, translating these findings requires a holistic strategy that navigates both the context-dependent signaling of specific myokines and their species-specific biological outcomes.

### 3.2. Protein Degradation and Systemic Aging

The autophagy–lysosome system (ALS) is one of the core mechanisms by which skeletal muscle maintains homeostasis and resists aging. FOXO3 and mTOR act as master regulators of autophagy, fine-tuning autophagic flux through mutual antagonism [144,145,146,147,148]. In *Drosophila*, overexpression of FOXO in flight muscles significantly enhances autophagosome and lysosome activity, thereby mitigating age-related protein aggregation and extending lifespan. In contrast, FOXO-deficient mutants show rapid proteostatic failure and accelerated muscle aging [149]. Consistently, single-nucleus profiling of skeletal muscle in aging primates reveals a downregulation of FOXO3 expression, further supporting its geroprotective role in skeletal muscle [150]. In naturally aging rats, skeletal muscle exhibits hyperactivation of mTOR and suppression of FOXO3a transcription. This combined dysregulation inhibits autophagic degradation, resulting in defective removal of damaged mitochondria and aggravated muscle atrophy. Notably, exercise intervention rescues muscle function by restoring ALS-mediated mitochondrial homeostasis [151]. Underscoring the critical role of autophagy. Muscle-specific knockout of *Atg7* (an essential autophagy effector enzyme) recapitulates these aging-related defects, including severe atrophy and mitochondrial dysfunction and is sufficient to shorten organismal lifespan [152,153].

In parallel with ALS, the ubiquitin-proteasome system (UPS) contributes to skeletal muscle proteostasis by degrading short-lived or misfolded proteins [154,155] (Figure 3C). The integrity of this system has also been identified as a key determinant of lifespan. The 26S proteasome subunit Rpt3 (PSMC4) is indispensable for protein degradation. Its muscle-specific knockout results in sever muscle wasting, and markedly decreased lifespan [156]. Consistently, Rpt3 deletion in muscle satellite cells restricts stem cell proliferation and increases its apoptosis. This exhaustion of the satellite cell pool compromises regenerative capacity, ultimately driving muscle atrophy and aging [157]. While the proteasome executes degradation, substrate specificity is governed by E3 ubiquitin ligases. A prime example is CHIP (carboxyl terminus of Hsc70-interacting protein), which targets misfolded proteins for proteasomal degradation. Min et al. found that its muscle-specific CHIP knockout precipitates proteotoxicity and oxidative stress, causing premature muscle atrophy, and reduced lifespan [158]. Another key regulator is UBR4 (Ubiquitin Protein Ligase E3 Component N-Recognin 4), which is typically upregulated during aging. In mice, muscle-specific knockout of UBR4 causes the accumulation of polyubiquitinated proteins, leading to decreased muscle contractility and impaired motor performance. Likewise, targeted knockdown in *Drosophila*, muscle mirrors these defects, aggravating protein aggregation and shortening lifespan [159].

In muscle, ALS and UPS not only maintain the structure and regenerative capacity of the muscle itself but also impact the aging of distal tissues. In aging and Alzheimer’s disease mouse models, activation of TFEB (transcription factor EB), a key node in the transcriptional regulation of ALS, in skeletal muscle alleviates neuroinflammation, enhances learning and memory, and delays neurodegeneration [160]. In spinal and bulbar muscular atrophy (SBMA) mouse models, TFEB function in skeletal muscle is impaired, leading to deficits in autophagy. This autophagic dysfunction exacerbates the accumulation of misfolded polyQ-AR proteins in both neurons and skeletal muscle cells, thereby inducing cytotoxicity and accelerating disease progression [161]. Remarkably, enhancement of TFEB expression exclusively in skeletal muscle significantly delays motor neuron degeneration and extends lifespan in SBMA mice [161]. In *Drosophila*, muscle-specific knockdown of the 20S proteasome core subunits Prosb1 or Prosb5 induces compensatory proteostasis preservation response in the central nervous system (CNS) during aging. This long-range signaling relies on muscle-secreted Amyrel, an amylase-related enzyme. Mimicking the upregulated expression of Amyrel in muscle tissue under stress conditions effectively reduces age-related accumulation of poly-ubiquitinated proteins in the brain and retina [162,163].

Collectively, ALS and UPS constitute the twin cornerstones of skeletal muscle proteostasis. These two proteolytic systems not only underpin the structural integrity and regenerative potential of muscle tissue but also serve as pivotal regulatory hubs that dictate the functional status of distal organs and the systemic aging trajectory. Therefore, decoding the intricate molecular networks and the initiating triggers that govern ALS and UPS dysfunction in muscle is crucial for elucidating the biological basis of tissue degeneration. Such insights will pave the way for the development of precision molecular interventions targeting muscle-specific protein degradation to counteract systemic aging.

### 3.3. Mitochondrial Regulation and Antioxidant Defense Mechanisms

Mitochondrial damage and impairment are thought to be key driving forces of muscle aging [164,165]. Thus, optimization of mitochondrial function within skeletal muscle may contribute to delaying the aging process (Figure 3D). In *Caenorhabditis elegans*, exercise -induced activation of AMPK signaling in body wall muscle promotes dynamic mitochondrial fission and fusion cycles, thereby delaying age-related decline in motor function [166]. In *Drosophila*, inhibition of PARP1, which becomes hyperactive with age, promotes mitochondrial biogenesis and extends lifespan [167]. Intriguingly, mild mitochondrial perturbations can also exert a geroprotective effect. In *Drosophila*, muscle-specific knockdown of mitochondrial fusion genes *Marf* and *Opa1* induces the loss of cristae structure. This mild stress improves *Drosophila* locomotor capacities and extends *Drosophila* lifespan via systemic metabolome reorganization [168]. Similarly, mild respiratory dysfunction originating in muscle—indued by NDUFS2 mutation or porin knockdown—promotes longevity through activation of mitophagy and systemic repression of insulin signaling [169].

The mitochondrial respiratory chain inevitably generates ROS during energy metabolism. The accumulation of ROS leads to oxidative stress, which is another contributor to muscle damage and aging. To counteract oxidative stress, cells have evolved intricate antioxidant defense systems. Nrf2 is a transcription factor that drives the expression of antioxidant enzymes to counteract oxidative stress. In mice, deletion of Nrf2 in skeletal muscle significantly exacerbates age-related mitochondrial oxidative stress, and consequently accelerates skeletal muscle aging [170]. AMPK enhances this antioxidant defense by indirectly activating Nrf2 [171,172]. Exercise training robustly activates the AMPK-Nrf2 signaling cascade, improving skeletal muscle adaptability to oxidative stress and promoting mitochondrial renewal and muscle repair [173,174]. In *Drosophila*, muscle-specific inhibition of AMPK significantly reduces stress tolerance and shortens lifespan, whereas similar interventions in neurons or adipose tissue do not produce such effects [175].

In conclusion, mitochondrial homeostasis is a key determinant in the regulation of systemic aging. While mitochondrial integrity is essential, subtle functional perturbations can confer anti-aging advantages via compensatory responses. Overt mitochondrial dysfunction directly precipitates age-related decline. Furthermore, the elevation of mitochondrially derived ROS acts as a distinct driver that accelerates muscle aging. To counteract this oxidative challenge, the endogenous antioxidant system, orchestrated by the AMPK/Nrf2 axis, serves as a critical protective barrier. Ultimately, a deeper understanding of the delicate balance between mitochondrial impairment, ROS-mediated damage and homeostatic defenses will provide foundational insights into the mechanistic underpinnings of both muscle-specific and systemic aging.

## 4. Multifaceted Intestinal Signaling in the Regulation of Systemic Aging

**Figure 4:**
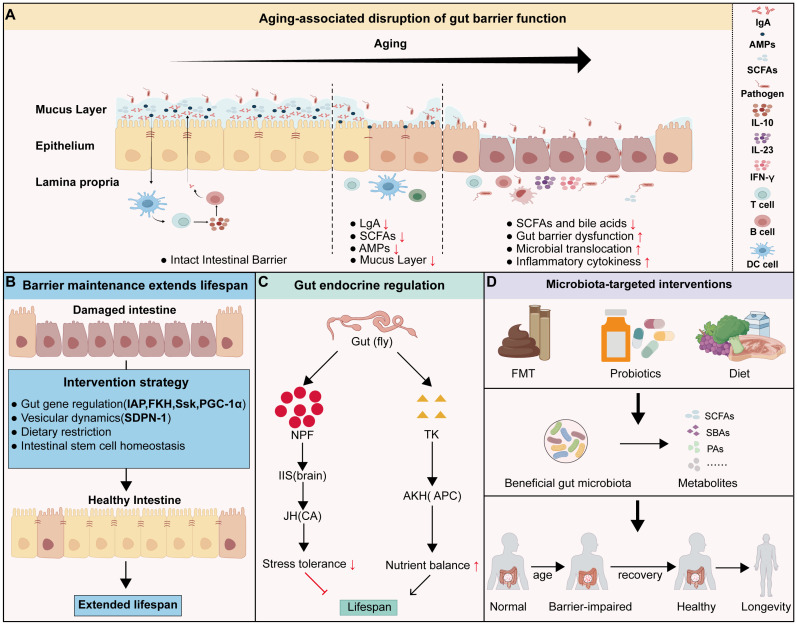
Functional deterioration of the gut during aging and gut-mediated regulation of lifespan: (**A**) Changes in the intestinal barrier with aging; (**B**) Maintenance of intestinal barrier homeostasis and lifespan extension through mechanisms such as gut gene modulation (Ssk, FKH, IAP, PGC-1α), vesicular dynamics (SDPN-1), dietary restriction, and stem cell homeostasis; (**C**) In *Drosophila*, gut-derived neuropeptides (NPF and TK) regulate lifespan via endocrine signaling pathways; (**D**) Interventions targeting the gut microbiota, including probiotics, fecal microbiota transplantation (FMT), and dietary modulation, promote the growth of beneficial microbial populations and the production of key metabolites (e.g., SCFAs, SBAs, PAs), which help repair the intestinal barrier and extend healthy longevity. Black arrows indicate the direction of the process or activation, while red T-bars indicate inhibition. Red upward and downward arrows indicate increased and decreased levels, respectively.

The intestine serves as a critical signaling hub that regulates systemic aging and organismal lifespan, extending its influence far beyond local digestive functions. Central to this signaling capacity is the integrity of the intestinal barrier. The intestinal barrier consists of epithelial cells, tight junctions, the mucus layer, and the mucosal immune system. This barrier effectively prevents the invasion of exogenous pathogens, regulates immune responses, and maintains microbial homeostasis within the gut (Figure 4A). However, aging progressively compromises this structural integrity and functional stability. Mechanistically, aging downregulates tight junction proteins, impairing the physical barrier [176]. Concurrently, mucosal immunosenescence occurs—characterized by diminished lymphocyte activity (B cells, T cells, DCs), reduced secretion efficiency, and elevated proinflammatory cytokines (e.g., IL-23, IFN-γ) [177]. This dual compromise of physical and immune barriers facilitates the translocation of pathogens and harmful metabolites, precipitating profound gut dysbiosis. Such microbial imbalance is a key driver of age-related pathologies, including metabolic syndrome, cardiovascular diseases, and neurodegenerative disorders [178] (Figure 4A). Consequently, preserving intestinal homeostasis represents a critical strategy for mitigating systemic aging.

### 4.1. Intestinal Barrier and Systemic Aging

Intestinal barrier dysfunction increases with age and is consistently linked to organismal decline across species [179,180,181]. The Smurf assay in *Drosophila* demonstrated that loss of intestinal barrier integrity is significantly correlated with the terminal phase of lifespan and serves as a reliable biomarker of impending death [182]. In mice, age-related increases in intestinal permeability allow proinflammatory factors such as lipopolysaccharide (LPS) to enter the circulation, thereby inducing chronic low-grade systemic inflammation and promoting systemic aging [183]. Similarly, aged cynomolgus monkeys exhibit pronounced intestinal barrier damage and elevated systemic inflammation [184,185].

Restoring intestinal barrier function has been shown to effectively delay aging through several convergent mechanisms (Figure 4B). First, maintaining metabolic and mitochondrial homeostasis is critical. Optimizing mitochondrial function (via PGC-1 and Complex III inhibition) or nutrient absorption (via FKH) preserves epithelial bioenergetics. This metabolic support directly strengthens barrier structure and prolongs lifespan [186,187,188,189]. Second, structural reinforcement plays a direct protective role. The transmembrane protein Snakeskin (Ssk) drives septate junction assembly, whereas Intestinal Alkaline Phosphatase (IAP) upregulates tight junction proteins. Loss of either protein accelerates aging, while their restoration extends lifespan. Notably, Ssk models suggest that barrier integrity dictates lifespan largely independently of microbial burden. Nevertheless, the gut microbiota remains a potent modulator of barrier stability [190,191,192]. Beyond these classical pathways, recent insights highlight vesicle trafficking as a novel regulatory node. Age-related upregulation of SDPN-1 disrupts the recycling of adherens junctions via RAB-10 inhibition. In contrast, SDPN-1 knockdown alleviates age-related loss of junction integrity and increased intestinal permeability [193].

These regulatory axes provide the mechanistic basis for established longevity interventions. For instance, dietary restriction (DR) enhances epithelial cellular fitness via dMyc, whereas methionine restriction (MR) reduces serum levels of IL-1β, TNF-α, and LPS, upregulates tight junction proteins such as occludin, claudin-1, and ZO-1. These systemic strategies extend lifespan by effectively coupling local epithelial homeostasis to the suppression of systemic inflammation [194,195,196,197].

### 4.2. Intestinal Stem Cell Repair and Regeneration

Intestinal stem cell (ISC) dysfunction is a critical hallmark of intestinal aging. The decline in ISC function leads to impaired epithelial regeneration and disruption of barrier architecture, thereby accelerating the aging process in the intestine and even throughout the entire system [198]. Aging ISCs acquire an inflammatory memory driven by chromatin remodeling and IFNγ-STAT1 signaling pathway. They propagate this signature to nascent epithelial cells, fueling systemic inflammation. This inflammatory environment then impairs ISC regeneration, creating a self-reinforcing cycle of intestinal decline [199,200,201]. Accordingly, restoring ISCs homeostasis represents a promising avenue for anti-aging interventions [202]. A prime example involves the differential modulation of UPR^ER^ (the endoplasmic reticulum-induced unfolded protein response). Chronic activation of the ER stress sensor PERK is maladaptive during aging. In *Drosophila*, ISC-specific inhibition of PERK suppresses age-related hyperproliferation and restores epithelial homeostasis, ultimately extending organismal lifespan [203]. Conversely, activating the adaptive UPR^ER^ pathway through Xbp1s (the spliced form of X-box binding protein 1) overexpression in ISCs enhances proteostasis, effectively preventing protein aggregations and delaying the onset of age-related intestinal dysplasia. By maintaining healthy tissue regeneration and barrier function, Xbp1s in the gut significantly extends organismal lifespan [204]. Beyond ER proteostasis, maintaining mitochondrial function is also critical. For instance, ISC-specific deletion of Fut2 (α1,2-fucosyltransferase) disrupts mitochondrial respiration and blocks mitophagy. The resulting ATP depletion and ROS accumulation compromise ISC stemness and accelerate aging [205]. Taken together, these studies establish ISC homeostasis as a central determinant of longevity, linking intestinal integrity to the retardation of systemic aging.

### 4.3. Gut-Derived Neuropeptides and Hormones

Gut-derived neuropeptides and hormones constitute a crucial mechanism through which the intestine regulates systemic aging (Figure 4C). The glucose-responsive hormone CCHamide-2 (CCHa2) and nutrient-sensitive neuropeptide F (NPF) function as primary somatic sensors that couple midgut metabolic status to the canonical insulin-signaling machinery. Specifically, in *Drosophila*, CCHa2 synthesized in the midgut stimulates insulin-producing cells (IPCs) to release insulin-like peptides (DILPs). Additionally, intestinal NPF suppression extends lifespan by downregulating insulin secretion and reducing systemic Juvenile Hormone (JH) titers [206,207]. Beyond insulin signaling, the gut regulates systemic aging by modulating metabolic trade-offs and energy homeostasis. In *Drosophila*, this regulation is orchestrated by distinct enteroendocrine hormones. The intestinal hormone Tachykinin (TK) acts as a pro-aging signal by driving lipid depletion in response to dietary protein, whereas Bursicon acts as a longevity factor by suppressing AKH-mediated lipolysis via neuronal LGR2 receptors [208,209]. Moreover, midgut senescence itself is antagonistically governed by allatostatin A (AstA) and diuretic hormone 31 (DH31). Age-related elevation of DH31 drives gut deterioration and shortens lifespan. In contrast, AstA acts as a critical anti-aging factor. Its silencing significantly reduces longevity. Notably, inhibiting DH31 signaling rescues these defects caused by AstA loss, thereby preserving gut integrity and extending survival [210].

Collectively, these findings demonstrate that the intestine exerts multifaceted control over aging, utilizing a diverse hormonal repertoire to modulate insulin signaling, regulate metabolic trade-offs, and actively maintain organismal homeostasis.

### 4.4. Gut Microbiota Balance

Although the gut microbiota does not directly constitute the intestinal tissue structure, it exerts profound influences on host metabolic homeostasis, immune regulation, and systemic inflammation [1,211] (Figure 4D). It has been proposed that host aging may originate from the decline in metabolic capacity of the gut microbiota. Thus, microbial remodeling represents an effective strategy for delaying aging [212]. Fecal microbiota transplantation (FMT) has been shown to reverse diverse aging phenotypes. It improves cognitive function and motor coordination, strengthens barrier integrity, and attenuates systemic inflammation in aged recipients [213,214]. Targeted probiotic supplementation further validates this connection. Specifically, supplementation with *Akkermansia muciniphila* (AKK, a probiotic) enhances barrier integrity by reducing serum lipopolysaccharide (LPS) levels and upregulating tight junction and mucin-encoding genes [215]. It also drives ISC-mediated barrier repair by activating Wnt/β-catenin signaling [216]. Similarly, probiotic interventions have demonstrated systemic benefits across the gut–muscle and gut–brain axes. In aged mice, *Lactobacillus paracasei* P62 (Lp) and *Bifidobacterium bifidum* P61 (Bb) supplementation reduces intestinal endotoxin and IL-1β levels. These changes correlates with decreased muscle atrophy marker (MuRF1/MAFbx) and increased hippocampal BDNF expression [217,218]. In parallel, *Lactobacillus casei Shirota* (LcS) supplementation ameliorates sarcopenia in senescence-accelerated models. This benefit is mediated by reshaping the gut microbiota, restoring short-chain fatty acid (SCFA) levels and suppressing pro-inflammatory taxa [219]. Dietary strategies reinforce these findings: fiber-rich diets in older adults and mice promote abundances of beneficial taxa (*Bifidobacterium*, *Lactobacillus*, and *Akkermansia*), leading to enhanced barrier function and reduced inflammation [220,221,222].

Microbial metabolites, particularly SCFAs, act as crucial mediators that alleviate age-related functional decline [223] (Table 2). An abundance of SCFA-producing bacteria is a hallmark of long-lived human populations. Persistent microbial dysbiosis reduces SCFA production, disrupting homeostasis and accelerating tissue aging [224,225]. Crucially, the protective influence of SCFAs extends far beyond the gut, preserving the functional integrity of distal organs. In skeletal muscle, they are required to prevent atrophy and maintain mass, as evidenced by the severe muscle loss in germ-free mice that is reversible with SCFA supplementation [226,227]. In the nervous system, SCFA preserve blood–brain barrier (BBB) integrity by maintaining tight junction [228]. Furthermore, SCFA exert significant neuroprotective effects by suppressing microglial activation and neuroinflammation. Mechanistically, by promoting histone acetylation and regulating complement pathway, SCFA treatment attenuates neuronal apoptosis, synaptic loss, and cognitive impairment across diverse models of neurodegeneration [229].

Taken together, these lines of evidence establish the intestine as a potent regulator of organismal lifespan. This systemic control is orchestrated by a multifaceted repertoire of mechanisms, including barrier mediated containment of inflammation, stem cell dependent regeneration, enteroendocrine calibration of systemic metabolism, and microbial modulation of host physiology. Consequently, targeting these distinct signaling axes represents a promising therapeutic strategy for combating systemic aging.

## 5. Protective Effects of Exercise Against Aging in Brain, Muscle, and Intestine

**Figure 5:**
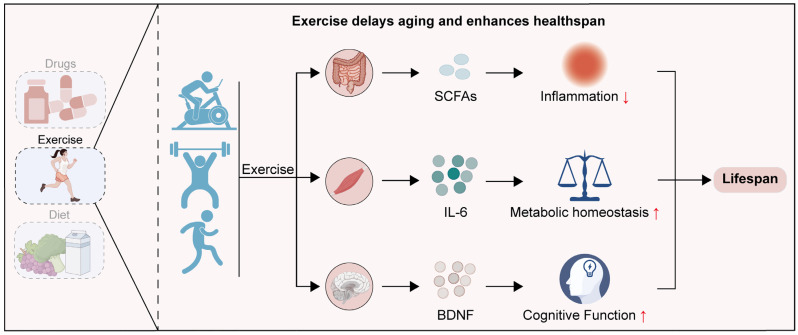
Exercise-mediated extension of lifespan via distinct organ-specific pathways. Black arrows indicate the direction of the process. Red upward and downward arrows indicate increased and decreased levels, respectively.

Given the pivotal roles of the brain, muscle, and intestine in orchestrating systemic aging, the age-related compromise of their regulatory functions inevitably precipitates organismal deterioration. This underscores the urgent need for interventions capable of restoring these compromised control hubs [244]. Among various intervention strategies, exercise has attracted widespread attention due to its high safety, broad applicability, and multi-target regulatory mechanisms [245,246,247] (Figure 5). A three-month regular exercise intervention in young mice (approximately 1 to 4 months of age) leads to sustained physiological benefits. This intervention enhances metabolic, cardiovascular, and muscular performance in later life, while significantly reducing systemic inflammation and frailty phenotypes during aging [248]. Epidemiological studies have demonstrated that high levels of physical activity during childhood and adolescence are positively associated with better aerobic fitness, lower incidence of obesity, and reduced cardiovascular risk in adulthood [249,250,251]. Meta-analyses further indicate that regular exercise helps adolescents manage body weight and improve metabolic parameters such as blood glucose, lipids, and blood pressure, thereby reducing the risk of chronic diseases in adulthood [252]. Notably, the mechanisms underlying the beneficial effects of exercise vary with age. Single-cell transcriptomic studies reveal age-specific effects of exercise. In young individuals, exercise primarily mitigates acute inflammatory injury. In contrast, in older individuals, it delays aging mainly by suppressing chronic inflammation and reprogramming the circadian clock through BMAL1-mediated pathways [253]. Although these pathways constitute a systemic framework for anti-aging, exercise-induced plasticity displays remarkable tissue-specific heterogeneity. The subsequent section will detail these unique adaptations within the brain, skeletal muscle, and the gut.

### 5.1. Exercise Delays Brain Aging

Regular aerobic exercise has been identified as an effective strategy for delaying brain aging [254]. A 12-month randomized controlled trial demonstrated that elderly individuals who underwent aerobic training showed an average increase of approximately 2% in hippocampal volume, along with significant improvements in spatial memory performance [255]. Imaging studies reveal that three months of moderate-intensity aerobic training increases hippocampal blood flow and metabolism in older adults. These physiological improvements are positively correlated with enhanced memory function [256]. Beyond the hippocampus, exercise also has a positive impact on the cortical structure. Longitudinal imaging studies found that sustained aerobic exercise increases the volume of gray and white matter in the prefrontal cortex of elderly individuals [257]. Functionally, fMRI-based studies have shown that after exercise training, elderly individuals exhibit significantly increased activation levels and functional connectivity of prefrontal networks during cognitive tasks [258,259]. A 9-year follow-up study further reported that elderly individuals with high physical activity levels exhibit less brain tissue atrophy [260].

Exercise-mediated protection against brain aging is mechanistically linked to enhanced levels of neurotrophic factors. Foremost among these is brain-derived neurotrophic factor (BDNF), which plays an indispensable role in maintaining synaptic plasticity and promoting neurogenesis. Beyond these structural benefits, BDNF also modulates glial immune responses and regulates cerebral energy homeostasis [261,262]. The therapeutic centrality of BDNF is underscored by direct intervention studies. For instance, a study on BDNF gene therapy confirmed that long-term expression of BDNF in the hippocampus continuously improves synaptic transmission in Alzheimer’s disease and partially reverses cognitive decline [263]. Similarly, intranasal delivery of exogenous mature BDNF significantly suppresses microglial activation and ameliorates aging-related memory deficits [264]. Crucially, physical exercise is a potent driver of endogenous BDNF. Long-term regular exercise significantly increases resting BDNF levels in elderly populations. Even a single session of acute aerobic exercise significantly elevates peripheral BDNF levels, with more pronounced effects seen in moderate to high-intensity exercise [265,266,267]. Additionally, resistance training and combined exercise also effectively increase BDNF levels. Their effects are comparable to those of aerobic exercise [268,269]. Regardless of the modality, this BDNF induction is orchestrated by key metabolic sensors. In an AD model, moderate-intensity running training activates the AMPK-PGC-1α-FNDC5-BDNF signaling axis [270]. This pathway also operates under chronic stress conditions, where exercise enhances BDNF via AMPK activation to reverse stress-induced memory impairment [271]. Alongside these neurotrophic effects, exercise also delays brain atrophy by improving cerebrovascular hemodynamics. In a mouse model of vascular dementia, long-term aerobic exercise enhances cerebral blood flow and hippocampal perfusion, and reduces white matter and hippocampal neuronal damage [272]. In an atherosclerosis (AS) mouse model, 10 weeks of aerobic exercise intervention not only significantly improves cerebral blood flow but also reduces excessive microglial activation and neuroinflammation in the hippocampus by inhibiting the IL-33/NF-κB signaling pathway [102]. Clinical studies in patients with amnestic mild cognitive impairment (aMCI) demonstrated that 1 year of high-intensity aerobic training significantly reduces carotid artery stiffness, enhances cerebral blood flow, and improves cognitive performance [273].

### 5.2. Exercise Delays Muscle Aging

Numerous studies have confirmed that regular exercise, particularly resistance training and aerobic exercise, effectively mitigates age-related muscle degeneration. For instance, 10 weeks of progressive resistance training has been shown to significantly improve muscle strength, gait speed, stair climbing ability, and daily living performance in older adults [274]. Similarly, endurance training enhances mitochondrial function and aerobic capacity. As a result, individuals who maintain a long-term exercise habit exhibit superior muscle fiber structure and overall function compared to sedentary peers [275].

Mechanistically, long-term regular exercise alleviates oxidative stress by suppressing the accumulation of 8-OHdG (a classic biomarker of DNA oxidative damage) and enhancing the activity of DNA repair and antioxidant proteins [276]. Distinct from these chronic adaptations, acute exercise induces a transient, massive surge in skeletal muscle IL-6 (>100-fold). While constitutive IL-6 elevation characterizes the pro-inflammatory aging phenotype, this acute, exercise-induced release functions distinctively to promote metabolic regulation and create an anti-inflammatory environment [277,278]. Furthermore, exercise is vital for maintaining regenerative potential [279]. In sarcopenia model, exercise activates the adiponectin/AdipoR1-AMPK signaling, which modulates downstream growth and survival pathways. These signaling changes improves mitochondrial function, inhibits muscle cell apoptosis, and promotes proliferation, ultimately enhancing muscle function and delaying aging [280,281].

### 5.3. Exercise Delays Intestinal Aging

Exercise has been shown to mitigate intestinal aging by remodeling the microbiota, promoting SCFA synthesis, and reinforcing the intestinal barrier. In terms of microbial composition and metabolites, Vijay et al. found that a 6-week exercise intervention in 78 elderly individuals increases SCFA production in the gut and significantly decreases inflammatory gene expression [230]. Consistently, a 12-week aerobic exercise intervention in ApoE-knockout mice significantly increases SCFA-producing bacteria such as *Rikenellaceae* and *Dubosiella*, while concurrently reducing the expression of TNF-α and IL-1β [231]. Beyond SCFAs, exercise also remodels eicosanoid metabolism. A recent multi-omics study revealed that long-term aerobic exercise mitigates intestinal senescence by restoring the abundance of *Akkermansia*. This microbial restoration suppresses the accumulation of the 14,15-DHET, a pro-inflammatory eicosanoid, thereby effectively countering age-related chronic inflammation [282]. Furthermore, exercise directly strengthens the intestinal barrier. In a mouse model of gut barrier dysfunction induced by repeated restraint stress, daily 30-min swimming sessions increase the levels of antimicrobial peptides (AMPs) in the small intestine and alleviate barrier dysfunction [283].

## 6. Conclusions and Future Perspectives

In summary, this review underscores a paradigm shift in aging research toward a “local-to-global” regulatory model. Specifically, we elucidate how the brain, skeletal muscle, and gut function as critical hubs that extend beyond local decline to actively drive systemic aging via long-range communication and systemic integration. However, a critical examination of the current literature reveals certain limitations in our scope. First, our analysis of brain-mediated regulation focused primarily on canonical structures such as the hypothalamus, hippocampus, prefrontal cortex and brainstem. Consequently, the modulatory roles of other brain regions remain to be fully elucidated. Second, regarding intervention strategies, this review was confined to physical exercise. While we acknowledge the critical influence of broader lifestyle determinants—such as dietary regimens and behavioral habits—these aspects fell outside the purview of the current discussion.

Regardless of the intervention modality, a fundamental gap persists: while we have delineated how tissue-specific genetic perturbations (e.g., within skeletal muscle) can elicit systemic anti-aging effects, the specific molecular transducers orchestrating this regulation remain elusive. The precise mechanisms by which local signals are propagated to distal targets are not fully understood. Although the proximal triggers (local genetic alterations) and distal outcomes (systemic lifespan extension) are well-characterized, the intermediate transduction machinery—comprising circulating factors or neural circuits that execute this “local-to-global” transmission—remains a “black box,” often obscured by the complexity of systemic signaling networks.

To advance from observing tissue-specific benefits to identifying systemic messengers, future research must prioritize characterizing the secretomes and signaling pathways of these organs. Emerging technologies now offer critical tools for this pursuit. For instance, in vivo tissue-specific labeling strategies facilitate the tracing of organ-derived proteins to their distal targets [284]. Complementarily, integrated single-cell transcriptomics can validate the cellular recipients of these signals [285]. However, distinguishing functional signaling molecules from circulating “biological noise” remains essential for identifying bona fide rejuvenation effectors.

Ultimately, this review underscores the therapeutic sufficiency of “local” interventions. By synthesizing evidence across the brain, muscle, and gut, we demonstrate that achieving systemic benefits does not require targeting the entire organism. Instead, preserving homeostasis in specific key tissues is sufficient to confer systemic anti-aging benefits. This insight suggests a refined therapeutic approach: future strategies should focus on the precise modulation of these key organs to induce the secretion of endogenous rejuvenation factors, thereby harnessing local targets to promote systemic health.

## Figures and Tables

**Table 1:** Myokines involved in delaying organismal aging.

Myokine	Target Tissues	Physiological Effects	Molecular Mechanisms	References
IL-6	Skeletal muscle, adipose tissue, liver, pancreas	Regulates metabolism and inflammation balance	JAK/STAT3; NF-κB	[110]
CLCF1	Skeletal muscle, bone tissue, heart	Promotes myogenesis and metabolic improvement	AKT/mTOR; STAT3	[117]
Irisin	Adipose tissue, skeletal muscle, heart, brain	Enhances mitochondrial function and neuroprotection	AMPK-PGC-1α-SIRT1	[118,119]
FGF21	Liver, adipose tissue, skeletal muscle	Improves insulin sensitivity and lipid metabolism	FGFR1c/β-Klotho; AMPK/SIRT1	[139]
Musclin	Skeletal muscle, bone tissue, heart	Maintains muscle metabolism and regeneration	NPR3-AKT-FOXO3	[129]
MSTN	Skeletal muscle, adipose tissue	Inhibits muscle growth; promotes sarcopenia	ActRIIB/Smad2/3	[133]
IL-15	Skeletal muscle, immune system	Preserves muscle mass and immune homeostasis	JAK/STAT5; AMPK	[140]
BAIBA	Skeletal muscle, adipose tissue	Enhances fatty acid oxidation; anti-inflammatory	AMPK-PPARα	[141]
Apelin	Skeletal muscle, cardiovascular system	Improves vascular and muscular function	APJ-AMPK-FOXO3	[142]
SPARC	Skeletal muscle, adipose tissue	Enhances insulin sensitivity and metabolic stability	AMPK-AKT	[143]

**Table 2:** The regulatory roles of SCFAs in aging.

Regulatory Mechanism	Subject	Effects of SCFAs	Molecular Mechanism	Outcome	Reference
Inflammation regulation	Older humans	Increased SCFA production and decreased systemic inflammatory cytokines,	GPR41/43 activation inhibited NF-κB signaling, reducing chronic inflammation.	Alleviated inflammaging	[230]
	*ApoE*^−/−^ mice	Improved intestinal inflammation and lipid metabolism.	SCFA upregulation suppressed NF-κB activation and reduced TNF-α and IL-1β expression.	Decreased inflammation	[231]
	HFHC-fed mice	Enrichment of anti-inflammatory microbiota (Rikenellaceae, Dubosiella), improving gut integrity.	GPR109A/GPR41 upregulation reduced inflammatory cytokine production.	Improved intestinal inflammation	[232]
	Human neutrophils	Attenuated LPS-induced inflammation; promoted apoptotic resolution.	GPR43 activation inhibited HDAC and NF-κB; induced caspase-dependent apoptosis.	Reduced inflammation, induced apoptosis	[233]
Glucose metabolism regulation	*FAF2*^−/−^ mice	Enhanced incretin secretion and improved glycemic stability.	GPR41/43 activation increased PYY and GLP-1 secretion via AMPK signaling.	Maintained glycemic stability	[234]
	Diabetic mice	Improved insulin sensitivity and reduced hepatic gluconeogenesis.	AMPK activation suppressed gluconeogenic enzymes.	Improved insulin sensitivity	[235,236]
Lipid metabolism regulation	Wild-type mice	Promoted lipid oxidation and thermogenesis.	LSD1 activation regulated thermogenic genes in BAT and scWAT.	Increased lipid oxidation	[237]
	HFD-fed mice	Reduced lipid accumulation and improved metabolic efficiency.	AMPK activation upregulated UCP-2, ACO, and CPT-1, suppressing lipogenesis.	Improved lipid metabolism	[238]
Autophagy regulation	Diabetic rats	Restored renal autophagy and reduced fibrosis markers.	AMPK/mTOR signaling activation enhanced autophagic flux.	Improved renal injury	[239]
	*db*/*db* mice	Reduced muscle inflammation and improved barrier function.	FFA2-PI3K/Akt/mTOR signaling regulated autophagy and mitigated muscle atrophy.	Improved muscle atrophy	[240]
	STC-1 cells	Enhanced LC3B-II accumulation and α-synuclein degradation.	Atg5-dependent autophagy and PI3K/Akt/mTOR activation alleviated PD pathology.	Attenuated PD progression	[241]
Other	PD mice	Improved neuronal function and reduced BBB damage.	Upregulation of Occludin and ZO-1 restored barrier integrity.	Improved neural injury	[242]
	Mice	Enhanced hippocampal LTP and memory performance.	PPARγ activation modulated synaptic plasticity and cognitive function.	Improved memory impairment	[243]

## Data Availability

No new data were created or analyzed in this study.
